# Fully Covered Self-Expandable Metal Stents for Treatment of Both Benign and Malignant Biliary Disorders

**DOI:** 10.1155/2012/498617

**Published:** 2012-06-14

**Authors:** Ahmed Abdel Samie, Lorenz Theilmann

**Affiliations:** Department of Gastroenterology, Pforzheim Hospital, 75175 Pforzheim, Germany

## Abstract

Transpapillary stents are increasingly being used for biliary strictures, whether benign or malignant. However, there are different stent types and available data is controversial. Recently, completely covered self-expandable metal stents (CSEMSs) have been proposed as an alternative therapeutic option in different biliary indications, including strictures of the distal bile duct, anastomotic stenosis after orthotopic liver transplantation, bile duct leaks, periampullary perforation following endoscopic sphincterotomy (ES), and postsphincterotomy bleeding. Despite the higher costs of these devices, fully covered self-expanding metal stents seem to be a suitable therapeutic option to relief biliary obstruction due to bile duct stenosis, regardless of the underlying cause.

## 1. Introduction

A multicenter trial comparing preoperative biliary drainage to early surgery for cancer of the pancreatic head showed that routine preoperative biliary drainage increased the rate of complications significantly [1]. The authors concluded that it should not be used routinely. Although drainage was primarily successful in 94%, there was a significant rate of cholangitis and a rate of 30% for the need of stent exchange leading to more readmissions. In addition, surgeons often report that they feel that anastomosis of the remaining biliary tract to the jejunal loop is more difficult and hampered because of the local inflammation caused by the stent. Nevertheless, in the trial above, plastic stents have been used. These stents have a narrow diameter which results in a high rate of stent occlusion over time with consequent cholangitis. This problem can partially be overcome using wide-bore stents such as self-expanding metal stents [2]. The disadvantage of these devices, however, is that once positioned they cannot be removed effortlessly without major damage to the biliary duct. Recently, completely covered self-expanding metal stents (CSEMSs) have become available making removal either endoscopically or during surgery easily possible. Accordingly, fully covered self-expandable metal stents have been lately used for the management of various malignant as well as benign biliary disorders, including strictures of the distal bile duct, anastomotic stenosis after orthotopic liver transplantation, and postsphincterotomy bleeding. 

Recent data demonstrated that biliary drainage can be achieved using fully covered self-expanding metal stents regardless of the underlying disease, be it benign or malignant. These stents were not associated with a higher rate of complication compared to noncovered self-expanding metal stents or plastic stents. In contrast to the latter a much lower rate of occlusion and subsequent cholangitis was observed. 

## 2. Fully Covered Self-Expandable Metal Stents for Treatment of Malignant Biliary Strictures

Pancreatic cancer is the most common cause of malignant biliary obstruction. Jaundice occurs in 70–90% of the patients during the course of the disease.

In the past, plastic stents were used in the first instance in the palliative care of these patients to relieve biliary obstruction; however, in this setting self-expandable metal stents have been increasingly used due to their long term patency. Compared with uncovered metal stents, covered metal stents have longer patency and a lower rate of tumor ingrowth, and have a higher rate of stent migration [3].

A randomized controlled trial comparing covered and uncovered metal stents in patients with malignant biliary obstruction showed a longer stent patency of covered (304 days) compared with uncovered stents (161 days) [4].

Preoperative biliary drainage using plastic stents in patients with pancreatic carcinoma has been shown to be inferior to early surgery and is associated with high rates of serious complications [1]. In the trial above, plastic stents have been used. These stents have a narrow diameter which results in a high rate of stent occlusion over time with consequent cholangitis.

In a study including 101 patients with pancreatic carcinoma, CSEMSs were placed for biliary decompression regardless of respectability. In 85 patients who did not undergo resection the mean patency duration of CSEMS was 5,5 months. Moreover, this treatment option seems to be a less costly intervention compared with other treatment options [5]. 

This is also in accordance with a recent report from Philadelphia, in which the authors recommend fully covered self-expanding stents as the initial intervention for biliary obstruction even if the surgical respectability status is uncertain [6]. 

The prolonged patency and extractability of these devices make them an attractive treatment option in patients with pancreatic carcinoma regardless of respectability.

## 3. CSEMS for Treatment of Benign Biliary Strictures

The most common causes of benign biliary stenosis are postsurgical and chronic pancreatitis [7]. 

Plastic stents have been traditionally used to treat benign biliary disorders because of their extractability and low costs. In a systemic review published in 2009 including 47 studies (1116 patients) comparing plastic stents (single or multiple) and uncovered self-expandable metal stents in management of benign biliary strictures, multiple plastic stents appeared to be more effective [8]. However, this data was collected before the CSEMS era. 

In a prospective study CSEMSs were placed in 44 patients with benign biliary strictures due to different etiologies (chronic pancreatitis in 19 patients, gall stones related strictures in 14 patients, post-liver-transplant stenosis in nine patients, autoimmune pancreatitis in one patient and primary sclerosing cholangitis in one patient). The median time of stent placement was 3.3 months. Resolution of strictures was documented in 34 patients (77%) after a median poststent removal follow-up time of 3.8 months [9]. However, in patients with chronic pancreatitis endoscopic therapy was less successful compared with the other etiologies (58% versus 92%). These results are in accordance with the results of older studies in which plastic stents were used to treat biliary strictures in this setting. Chronic pancreatitis, especially calcific pancreatitis, seems to be less responsive to endoscopic therapy with a higher recurrence rate [7].

## 4. CSEMS for Treatment of Anastomosis Stenosis Following Liver Transplantation

The most common complication after liver transplantation is anastomotic stricture with an incidence of up to 15% in deceased donor liver transplantation [10], followed by biliary leakage. 

In a small series, including 11 patients with complications after liver transplantation, eight of whom with biliary stenosis and three with biliary leakage, placement of CSEMS was successful in avoiding hepaticojejunostomies in six from nine patients [11].

In a recently published paper which included 54 consecutive patients with biliary complications following orthotopic liver transplantation CSEMS proved to be successful in patients in whom standard endoscopic therapy failed. The authors concluded that metal stents should not be used as the primary treatment modality; however, after failure of conventional endoscopic therapy, temporary placement of CSEMS solved biliary problems in almost three-quarters of the patients [12].

 In this setting, however, stent migration seems to be a relevant problem occurring in between 30 and 50% of the cases, although presenting with no clinical consequences in most patients [11, 13]. 

We hypothesise that stent dislocation in anastomotic strictures following liver transplantation represents an indicator of the effectiveness of this therapy, as the currently patent bile duct cannot hold the stent any more in position. Furthermore, there were no serious complications associated with spontaneous stent migration. In our opinion, in anastomotic stricture complicating liver transplantation scheduled ERC by no later than eight weeks after stent implantation should be performed, in order that early/incomplete stent dislocation/migration can be detected, and if necessary correction of stent position or removal of dislocated stent can be achieved. 

New development, like anchoring flap at the proximal end of the stent may be an option as well to prevent this complication [14].

Not only in anastomotic stenosis following liver transplantation and distal biliary strictures, but also in hilar strictures CSEMS appears to be a therapeutic alternative without fear of occlusion of the contralateral hepatic duct, as shown in a recent paper [15]. Nevertheless, because of the very small number of cases, further evaluation is necessary.

## 5. CSEMS for Treatment of Postsphincterotomy Bleeding

Bleeding is one of the most common complications following endoscopic sphincterotomy.The incidence of postsphincterotomy bleeding reported in the literature widely varies because of differences in definition and may reach up to 10% [16].

Established risk factors of bleeding include uncorrected coagulopathy at the time of endoscopy, use of anticoagulants within three days prior to the procedure, and acute cholangitis. In addition, the presence of a periampullary diverticulum, the use of precut technique, and low endoscopist experience may increase the risk of bleeding [17]. 

As most of the bleeding episodes stop spontaneously, endoscopic therapy is preserved for endoscopically significant immediate bleeding and clinically relevant delayed bleeding.

Endoscopic therapy includes injection, thermal, and mechanical therapy using balloon tamponade or endoclips. Injection of diluted epinephrine (1 : 10,000) in and around the sphincterotomy site is the most commonly used method, whereas the amount of injected solution may vary from 0,5 to 30 mL [18]. 

Nevertheless, application of endoscopic therapy may be technically very demanding due to failure of exact localization of the bleeding site during severe hemorrhage and difficulty in maneuvering instruments through a side-view endoscope. Furthermore, the risk of pancreatitis may increase if endoscopic combination therapy is applied [19].

In a recent retrospective analysis including 11 patients, hemostasis was achieved in all patients using CSEMS after failure of other measures. The mean duration of stent placement was 8,2 days and all stents were successfully removed endoscopically [20]. In another case series, including five patients CESMS were effective to control bleeding in all patients. The stents were removed within eight weeks in three patients and migrated spontaneously without clinical sequelae in two patients [21].

A further advantage of this treatment modality is the simultaneous and effective drainage of the bile duct, especially if occluded with blood clots. 

## 6. CSEMS for Treatment of Biliary Leaks

Biliary leak is not an uncommon complication following hepatobiliary surgery. Leaks are treated with endoscopic sphincterotomy with or without stenting reducing the intraductal pressure and thus diverting the flow form the leaking site. Plastic stents have been used traditionally for this indication; however, CSEMS may provide a further effective therapeutic option. The large diameter of these stents allows an effective drainage away for the leakage site. Furthermore, placing the stent across the injured site may help to seal it [7].

In a retrospective analysis including 13 patients with complex biliary leaks (eight following cholecystectomy and five after liver transplantation) CSEMSs with anchoring fins were temporarily placed. Successful resolution of the leaks was achieved in all patients, though two patients developed strictures after stent removal. Furthermore, this treatment modality was associated with bile duct ulcerations and de novo choledocholithiasis [22].

## 7. CSEMS for Treatment of ERCP-Associated Perforations

ERCP-associated perforation may be due to endoscope passage leading to lateral duodenal wall injury (type I), periampullary following endoscopic sphincterotomy (type II), and ductal (type III) resulting from guide wire insertion.

Type I perforations are usually large and require immediate surgical treatment. 

On the other hand, type II perforations due to guide wire or basket insertion are typically small, tend to heal spontaneously, and are generally treated conservatively [23].

The management of periampullary perforations remains controversial. Some authors recommend immediate endoscopic treatment with diversion of bile from the perforation site using bile duct stents or nasobiliary tubes once retroperitoneal perforation is recognized during the procedure [23]. CSEMS represents a new endoscopic therapeutic option sealing the perforation site and permitting free bile flow into the duodenum. 

## 8. Removal of CSEMS

So far, only plastic stents could be removed safely endoscopically if necessary. The disadvantage of these stents, however, is the small internal diameter which predisposes to occlusion by biliary sludge. With the availability of fully covered self-expanding stents this short coming has been overcome. 

In a multicenter study, including 37 patients removal attempts of the CSEMS were successful in all cases [24]. The endoscopic feasibility and safety of stent removal were also documented by other authors [25]. 

## 9. Complications

Cholecystitis is not an uncommon complication following placement of CSEMS as the outlet of the cystic duct into the common bile duct can be blocked by the stent. We suggest that this complication should be avoided by using a stent length with the upper end distal to the cystic duct outlet. In one study, this complication occurred in 20% of cases if the CSEMS covered the cystic duct. A gallbladder stent (seven French transpapillary pigtail gallbladder stent) was effective in reducing the risk of developing cholecystitis after CSEMS placement [26].

Stent migration represents a frequent complication of CSEMS when placed for treatment of benign biliary strictures and has already been discussed above. Recently new stents were developed (anchoring flap) to decrease stent migration.

## 10. Conclusion 

Despite the higher costs of these devices, fully covered self-expandable metal stents are suitable to relief biliary obstruction due to bile duct stenosis, regardless of the underlying cause. Stent length should be chosen as short as possible to avoid any alteration of the proximal common bile duct. The internal diameter of 8–10 mm of these stents ascertains sufficient bile flow, if bile is very viscous because of previous biliary obstruction. Removal of the stents can be achieved smoothly in all cases if desired. 

CSEMS are also an effective treatment option in biliary leaks, type II perforation following ES, and severe postsphincterotomy bleeding, not controlled by other measures. 

Prospective multicenter trials should be carried out to evaluate further the effectiveness of CSEMS in benign and malignant biliary disorders.

## 11. Key Issues

The Key issues include the following.

- Fully covered self-expandable metal stents have been lately used for the management of various malignant as well benign biliary disorders.
- Recent reports demonstrated that biliary drainage can be achieved using fully covered self-expanding metal stents regardless of the underlying disease, be it benign or malignant.
- CSEMS can be also applied to treat biliary leaks following hepatobiliary surgery and type II perforation due to ES.
- CSEMS is an effective therapeutic option for treatment of postsphincterotomy bleeding not controlled by other measures.
- CSEMS can be removed easily either endoscopically or during surgery.
- Stent migration seems to be a relevant problem in benign indications occurring in up to 50% of the cases.
- Cholecystitis is not an uncommon complication following placement of CSEMS as the outlet of the cystic duct into the common bile duct can be blocked by the stent.
